# Association between triglyceride glucose-body mass index and 365-day mortality in patients with critical coronary heart disease

**DOI:** 10.3389/fendo.2025.1513898

**Published:** 2025-04-04

**Authors:** Jing Tian, Yan Dong, Zhongping Xu, Jin Ke, Hongyang Xu

**Affiliations:** Department of Critical Care Medicine, The Affiliated Wuxi People’s Hospital of Nanjing Medical University, Wuxi People’s Hospital, Wuxi Medical Center, Nanjing Medical University, Wuxi, Jiangsu, China

**Keywords:** coronary heart disease (CHD), triglyceride glucose-body mass index (TyG-BMI), insulin resistance, prognosis, mortality

## Abstract

**Objectives:**

The aim of this study was to analyze the association between TyG-BMI and 365-day mortality in critically ill patients with CHD.

**Methods:**

Patient data were extracted from the MIMIC-IV database. All patients were categorized into 3 groups based on TyG-BMI index: Low TyG-BMI index group, Medium TyG-BMI index group, and High TyG-BMI index group. Outcomes included primary and secondary outcomes, with the primary outcome being 365-day mortality and the secondary outcomes being hospital survival, intensive care unit (ICU) survival, and 28-day, 90-day, and 180-day mortality. The Kaplan-Meier survival curves were used to compare the outcomes of the three groups. The relationship between TyG-BMI index and 365-day mortality was assessed using multivariate Cox proportional risk regression models and restricted cubic spline curves (RCS).

**Results:**

889 critically ill patients with CHD were analyzed. Among them, 600 (67.50%) were male patients with a mean age of 68.37 years and 289 (32.50%) were female patients with a mean age of 73.91 years. Patients with a medium TyG-BMI index had the best 365-day prognostic outcome and the highest survival rate compared with patients in the Low and High TyG-BMI index groups [201 (67.68%) vs. 166 (56.08%), 188 (63.51%); P=0.013]. After fully adjusted modeling analysis, the hazard ratio (HR) for 365-day mortality was found to be 0.71 (95% CI 0.54-0.93, P=0.012) for the Medium TyG-BMI index group. Meanwhile, RCS analysis showed an L-shaped relationship between TyG-BMI index and 365-day mortality.

**Conclusions:**

The TyG-BMI index is significantly associated with 365-day mortality in patients with severe CHD.

## Introduction

The development and progression of coronary heart disease (CHD) is closely related to atherosclerosis, in which inflammation, vascular endothelial dysfunction, hypertension, dyslipidemia, and insulin resistance (IR) can increase the risk of atherosclerosis and thus affect the prevalence of CHD (1, 2). Presently, CHD and its clinical symptoms hold the record for the highest global morbidity and mortality rates, responsible for 32% of all deaths, ranking at the top in the worldwide disease burden, and exerting significant strain on national health finances (3–5). Prompt identification and prompt handling of CHD are crucial for directing medical professionals and lessening the worldwide impact of cardiovascular conditions.

IR, defined as reduced or impaired insulin sensitivity in insulin-dependent tissues or organs manifested by impaired glucose uptake and oxidation (6), is an important risk factor for the development of type 2 diabetes and CHD. It is part of a spectrum of cardiovascular metabolic abnormalities, often referred to as IR syndrome or metabolic syndrome, which accelerates atherosclerosis and thus the prognosis of patients with CHD (7). Overweight and obesity contribute to the development of cardiovascular disease (CVD), especially CHD (8). And, obesity is also recognized as an independent risk factor for CVD. Metabolic syndrome is closely related to CVD, including CHD, and centripetal obesity is an important component of metabolic syndrome (9).

In the absence of an effective method to accurately assess IR, the triglyceride-glucose (TyG) index, based on fasting triglyceride (TG) and blood glucose levels, has become a surrogate marker that is widely used in CVD (10–13). In contrast, the triglyceride glucose body mass index (TyG-BMI), which combines obesity measurements with the TyG index, has high sensitivity and specificity for recognizing IR, and both metrics have been validated in IR (14–16) However, the interaction between these two indices is largely underexplored in critically ill patients with CHD.

Therefore, the aim of our study was to investigate the relationship between TyG-BMI index and mortality outcomes in critically ill patients with CHD.

## Methods

### Source of data

This study is a retrospective analysis of data extracted from a large, publicly available critical care database, the Medical Information Marketplace for Critical Care IV (MIMIC-IV, version 2.2).The MIMIC-IV database, which has a number of enhancements to MIMIC-III, including data updates and some table reconstruction that collects clinical data on more than 190,000 admissions and more than 450,000 hospitalizations recorded at Beth Israel Deaconess Medical Center (BIDMC, Boston, Massachusetts, USA) from 2008 to 2019. The database records detailed information on patient demographics, laboratory tests, medications, vital signs, surgical procedures, disease diagnoses, medication management, and follow-up survival status. To gain access to the data, we researched the National Institutes of Health (NIH) training curriculum for the protection of human research participants and passed the Collaborating Institutions Training Program test. Informed consent was waived because the database did not contain protected information and patients were anonymous.

### Data extraction

Navicat Premium (version 16.1.15) was used to extract data using Structured Query Language (SQL). Clinical data for each patient were extracted, including demographic information (age, gender, height, weight); past history (hypertension, type 1 diabetes mellitus (T1DM), type 2 diabetes mellitus (T2DM), chronic kidney disease (CKD), acute myocardial infarction (AMI), stroke); disease severity scores (sequential organ failure assessment (SOFA), acute physiology score III (Aps iii), systemic inflammatory response syndrome (Sirs), simplified acute physiology score II (Saps ii), oxford acute severity of illness score (Oasis)); vital signs (heart rate (HR), systolic blood pressure (SBP), diastolic blood pressure (DBP)); medications (angiotensin II coenzyme inhibitor (ACEI), angiotensin II receptor blocker (ARB), calcium channel blocker (CCB)); laboratory data (white blood cell count (WBC), red blood cell count (RBC), glucose, TG, total cholesterol (TC), bilirubin, high density lipoprotein cholesterol (HDL-C), low density lipoprotein cholesterol (LDL-C), creatinine, troponin T (TnT)); management (mechanical ventilation (MV) use, days of MV, continuous renal replacement therapy (CRRT) use); length of stay (length of hospitalization and length of ICU) and outcomes (hospital survival, ICU survival, 28-day mortality, 90-day mortality, 180-day mortality, 365-day mortality). All blood indices were measured for the first time after the patient’s admission to the ICU. Variables with more than 20% missing values were excluded. Variables with less than 20% missing values were filled in using multiple interpolation.

### Study design and population

**Figure 1:**
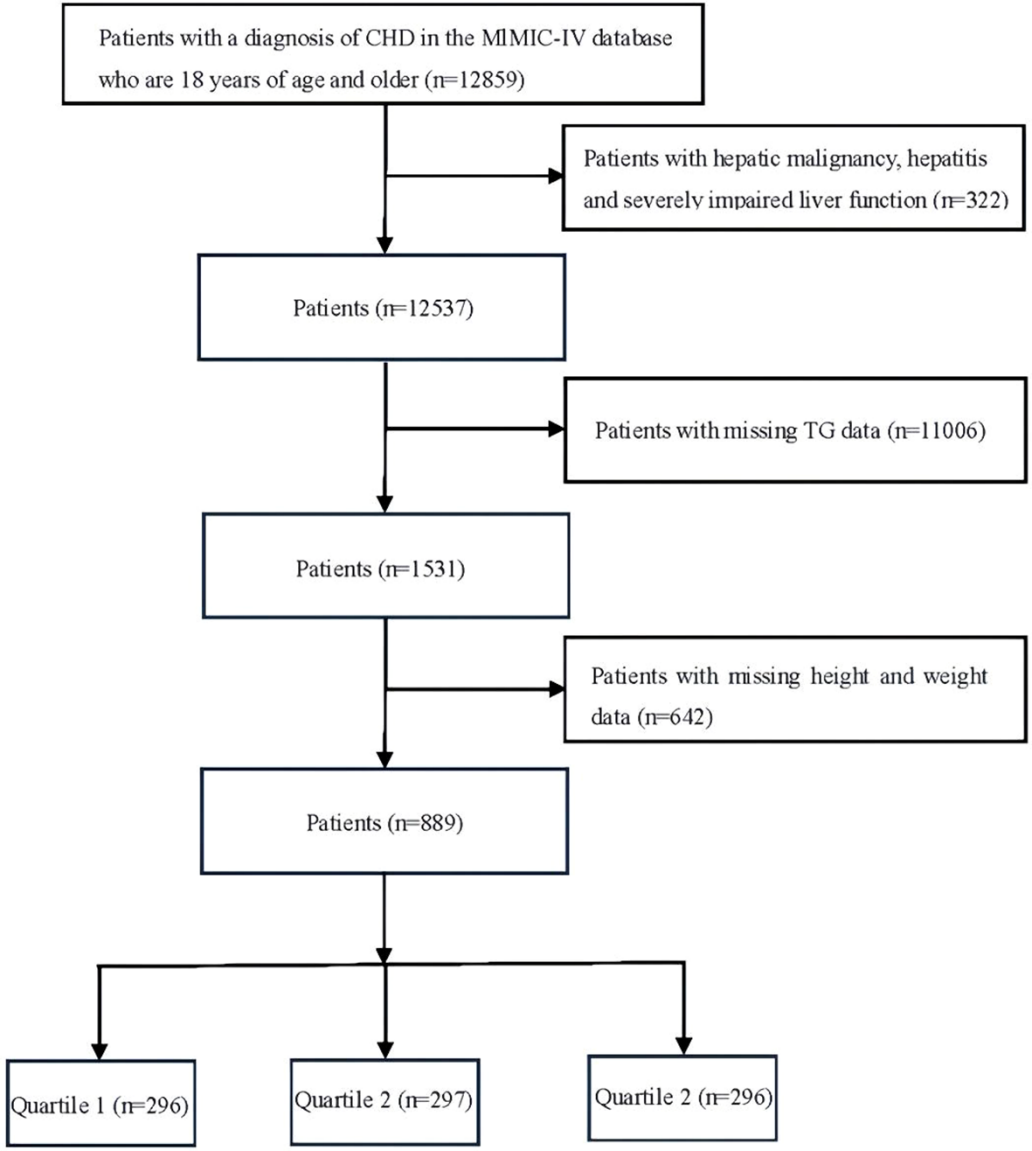
Flowchart of the selection of patients. Quartile 1, Low TyG-BMI index (<235.33) Quartile 2, Medium TyG-BMI index (235.33-294.40) Quartile 3, High TyG-BMI index (>294.40).

In this study, 12859 adult patients with severe CHD were retrieved from the MIMIC IV database. Exclusion criteria: 1. Patients with hepatic malignancy, severe hepatitis, and hepatic insufficiency were excluded, 2. Patients with missing TG data, and 3. Patients with missing height and weight data. After the exclusion criteria, 889 patients were finally included and grouped into three quartiles based on TyG-BMI index [TyG-BMI = (ln[fasting glucose (mg/dl) × fasting TG (mg/dl)]/2) * BMI (kg/m^2^)], and divided into three groups Quartile 1 [Low TyG-BMI index (<235.33)], Quartile 2 [Medium TyG-BMI index (235.33-294.40)], and Quartile 3 [High TyG-BMI index (>294.40)]. Specific data are shown in **Figure 1**.

### Outcomes

Primary outcome is the patient’s 365-day prognosis. Secondary outcomes are the patients’ hospital survival, ICU survival, and 28-day,90-day, and 180-day prognosis.

### Calculation of TyG-BMI

The TyG index was calculated as ln[fasting glucose (mg/dl) × fasting TG (mg/dl)/2]. BMI was calculated as body weight (kg)/height^2^ (m). TyG-BMI index was computed according the equation: TyG index × BMI (16).

### Statistical analysis

Initially, the normality of continuous variables was assessed, followed by the application of Student t-tests and one-way ANOVAs to pinpoint data adhering to a normal distribution, subsequently represented as mean ± standard deviation (SD). Data that did not follow a normal distribution underwent testing through the Wilcoxon rank-sum test and were presented as a median value with an interquartile range (IQR). To examine categorical variables represented in absolute figures and percentages, Chi-square or Fisher exact tests were employed.

The prognosis of patients was compared by stratifying by TyG-BMI index using Kaplan-Meier survival curves. Multivariate Cox proportional risk regression models were used to explore the relationship between TyG-BMI and 365-day mortality. A total of four models were developed (with low TyG-BMI index as the reference). Model 1 was unadjusted for variables. Model 2 was adjusted for sex and age, and Model 3 was adjusted for Age, Sex, Hypertension, T2DM, T1DM, CKD, AMI, Sroke, WBC, RBC, TC, HDL-C, LDL-C, Bilirubin, Creatinine, TnT, HR, SBP and DBP. Model 4 was adjusted for Age, Sex, Hypertension, T2DM, T1DM, CKD, AMI, Sroke, WBC, RBC, TC, HDL-C, LDL-C, Bilirubin, Creatinine, TnT, HR, SBP, DBP, Sofa, Apsiii, Sirs, Sapsii and Oasis. The TyG-BMI index was also analyzed using restricted cubic spline (RCS) as a continuous variable to elucidate the correlation of dose effects with the risk of major outcome events. In addition, stratified analyses were performed to assess the consistency of the prognostic value of the TyG-BMI index with the primary outcome. The analyses took into account sex, age, and the presence of comorbidities.

All data analyses were processed using R software (version 4.3.2, R Foundation for Statistical Computing, Austria) for all statistical analyses, and P < 0.05 was considered statistically significant.

## Results

### Baseline characteristics of study individuals

A total of 889 critically ill patients with CHD from MIMIC IV data were included in this study, of which 600 (67.50%) were male patients with a mean age of 68.37 years and 289 (32.50%) were female patients with a mean age of 73.91 years. The patients were categorized into three groups based on the tertiles of TyG-BMI index, Low TyG-BMI index (n=296, TyG-BMI: <235.33), Medium TyG-BMI index (n=297, TyG-BM: 235.33-294.40) and High TyG-BMI index (n=296, TyG-BMI: >294.40).

In **Table 1**, patients in the High TyG-BMI group were younger [67 (60, 74) years vs. 77 (66, 84) years, 71 (62, 79) years; p<0.001], had a higher BMI [36 (33, 40) kg/m2 vs. 23 (21, 25) kg/m2, 29 (27, 31) kg/m2; P<0.001], and most of them had T2DM [148 (50.00%) vs. 77 (26.01%), 104 (35.02%); P<0.001]compared to the Low and Medium TyG-BMI groups. Based on disease severity scores, patients with high TyG-BMI index had higher SOFA [6.0 (3.0, 10.0) vs. 4.0 (2.8, 7.0), 4.0 (2.0, 8.0); P<0.001], Aps iii [48 (34, 66) vs. 44 (34, 56), 42 (30, 55); P=0.013], Sirs [3.00 (2.00, 3.00) vs. 3.00 (2.00, 3.00), 3.00 (2.00, 3.00);P=0.035] and Saps ii scores [41 (29, 51) vs. 39 (31, 47), 35 (27, 46); P=0.006], and laboratory markers such as erythrocytes, TG [160 (110, 257) mg/dL vs. 90 (70, 126) mg/dL, 118 (87, 167) mg/dL; P<0.001], Creatinine [1.20 (0.90, 1.70) mg/dL vs. 1.10 (0.80, 1.60) mg/dL, 1.10 (0.90, 1.40) mg/dL; P=0.006], HDL-C [36 (31, 40) mg/dL vs. 41 (36, 51) mg/dL, 39 (34, 47) mg/dL; P<0.001], LDL-C and TnT [1.11 (0.21, 2.13) ng/mL vs. 0.77 (0.17, 1.53) ng/mL, 0.96 (0.21, 2.63) ng/mL; P=0.014] were also higher in these patients.

**Table 1:** Baseline characteristics of patients grouped according to TyG-BMI index.

	Low TyG-BMI index (n=296)	Medium TyG-BMI index (n=297)	High TyG-BMI index (n=296)	p-value
Age, years	77 (66, 84)	71 (62, 79)	67 (60, 74)	<0.001
Sex, %				0.582
Female	103 (34.80%)	94 (31.65%)	92 (31.08%)	
Male	193 (65.20%)	203 (68.35%)	204 (68.92%)	
BMI, kg/m^2^	23 (21, 25)	29 (27, 31)	36 (33, 40)	<0.001
Comorbidities, %				
Hypertension				0.497
0	176 (59.46%)	165 (55.56%)	163 (55.07%)	
1	120 (40.54%)	132 (44.44%)	133 (44.93%)	
T2DM				<0.001
0	219 (73.99%)	193 (64.98%)	148 (50.00%)	
1	77 (26.01%)	104 (35.02%)	148 (50.00%)	
T1DM				0.171
0	285 (96.28%)	292 (98.32%)	291 (98.31%)	
1	11 (3.72%)	5 (1.68%)	5 (1.69%)	
CKD				0.064
0	217 (73.31%)	239 (80.47%)	217 (73.31%)	
1	79 (26.69%)	58 (19.53%)	79 (26.69%)	
AMI				0.022
0	207 (69.93%)	176 (59.26%)	196 (66.22%)	
1	89 (30.07%)	121 (40.74%)	100 (33.78%)	
Sroke				0.041
0	250 (84.46%)	271 (91.25%)	259 (87.50%)	
1	46 (15.54%)	26 (8.75%)	37 (12.50%)	
Severity of illness				
Sofa	4.0 (2.8, 7.0)	4.0 (2.0, 8.0)	6.0 (3.0, 10.0)	<0.001
Aps iii	44 (34, 56)	42 (30, 55)	48 (34, 66)	0.013
Sirs	3.00 (2.00, 3.00)	3.00 (2.00, 3.00)	3.00 (2.00, 3.00)	0.035
Saps ii	39 (31, 47)	35 (27, 46)	41 (29, 51)	0.006
Oasis	33 (27, 40)	33 (26, 39)	35 (27, 43)	0.060
Vital signs				
HR, bpm	86 (74, 98)	84 (72, 99)	86 (74, 100)	0.700
SBP, mm Hg	122 (108, 138)	124 (108, 142)	120 (105, 139)	0.131
DBP, mm Hg	68 (55, 82)	70 (59, 81)	68 (57, 81)	0.358
Laboratory tests				
WBC, K/uL	11 (8, 15)	12 (9, 16)	13 (10, 18)	<0.001
RBC, K/uL	3.64 (2.96, 4.19)	3.93 (3.26, 4.44)	3.87 (3.26, 4.47)	<0.001
Glucose, mg/dL	123 (101, 160)	135 (109, 185)	165 (131, 226)	<0.001
TG, mg/dL	90 (70, 126)	118 (87, 167)	160 (110, 257)	<0.001
TC, mg/dL	129 (114, 151)	133 (119, 166)	131 (118, 155)	0.049
Bilirubin, mg/dL	0.70 (0.50, 0.90)	0.67 (0.50, 0.90)	0.70 (0.50, 1.00)	0.558
HDL-C, mg/dL	41 (36, 51)	39 (34, 47)	36 (31, 40)	<0.001
LDL-C, mg/dL	65 (54, 82)	68 (59, 99)	67 (59, 85)	0.012
Creatinine, mg/dL	1.10 (0.80, 1.60)	1.10 (0.90, 1.40)	1.20 (0.90, 1.70)	0.006
TnT, ng/mL	0.77 (0.17, 1.53)	0.96 (0.21, 2.63)	1.11 (0.21, 2.13)	0.014

BMI, Body mass index; T2DM, Diabetes mellitus type 2; T1DM, Diabetes mellitus type 1; CKD, Chronic kidney disease; AMI, Acute myocardial infarction; Sofa, Sequential organ failure assessment; Aps iii, Acute physiology score III; Sirs, Systemic inflammatory response syndrome; Saps ii, Simplified acute physiology score II; Oasis, Oxford acute severity of illness score; HR, Heart rate; SBP, Systolic blood pressure; DBP, Diastolic blood pressure; WBC, White blood cell count; RBC, Red blood cell count; TG, Triglyceride; TC, Total cholesterol; HDL-C, High density lipoprotein cholesterol; LDL-C, Low density lipoprotein cholesterol; TnT, Troponin T.

### Clinical management of patients

Compared with patients in the Low TyG-BMI index and Medium TyG-BMI index groups, patients in the High TyG-BMI index group had essentially similar rates of ACEI and ARB medication use, but higher rates of mechanical ventilation [188 (63.51%) vs. 159 (53.72%), 146 (49.16%); P=0.002]and CRRT [53 (17.91%) vs. 27 (9.12%), 30 (10.10%); P=0.002]application, and prolonged duration of mechanical ventilation support[26 (0, 149) days vs. 6 (0, 54) days, 0 (0, 63) days; P<0.001]. In addition to longer lengths of ICU stays [5 (2, 13) days vs. 4 (2, 9) days, 4 (2, 8) days; P=0.009] for these patients. See **Table 2** for specific data.

**Table 2:** Clinical management of critically ill patients with CHD.

	Low TyG-BMI index (n=296)	Medium TyG-BMI index (n=297)	High TyG-BMI index (n=296)	p-value
Medication, %				
ACEI				0.059
0	290 (97.97%)	282 (94.95%)	279 (94.26%)	
1	6 (2.03%)	15 (5.05%)	17 (5.74%)	
ARB				0.059
0	290 (97.97%)	282 (94.95%)	279 (94.26%)	
1	6 (2.03%)	15 (5.05%)	17 (5.74%)	
CCB use				0.003
0	280 (94.59%)	257 (86.53%)	260 (87.84%)	
1	16 (5.41%)	40 (13.47%)	36 (12.16%)	
MV use, %				0.002
0	137 (46.28%)	151 (50.84%)	108 (36.49%)	
1	159 (53.72%)	146 (49.16%)	188 (63.51%)	
MV time, days	6 (0, 54)	0 (0, 63)	26 (0, 149)	<0.001
CRRT use, %				0.002
0	269 (90.88%)	267 (89.90%)	243 (82.09%)	
1	27 (9.12%)	30 (10.10%)	53 (17.91%)	
Length of hospitalization, days	11 (6, 21)	10 (5, 17)	11 (6, 22)	0.142
Length of ICU, days	4 (2, 9)	4 (2, 8)	5 (2, 13)	0.009

ACEI, Angiotensin II coenzyme inhibitor; ARB, Angiotensin II receptor blocker; CCB, Calcium channel blocker.

MV, Mechanical ventilation; CRRT, Continuous renal replacement therapy; ICU, Intensive Care Unit.

### Secondary and primary outcomings

**Figure 2:**
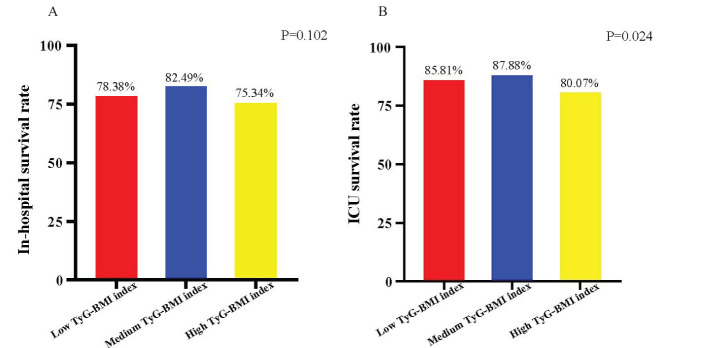
Survival rates associated with patients with different TyG-BMI index. **(A)** In-hospital survival rate of patients with different TyG-BMI index. **(B)** ICU survival rate of patients with different TyG-BMI index.

**Figure 3:**
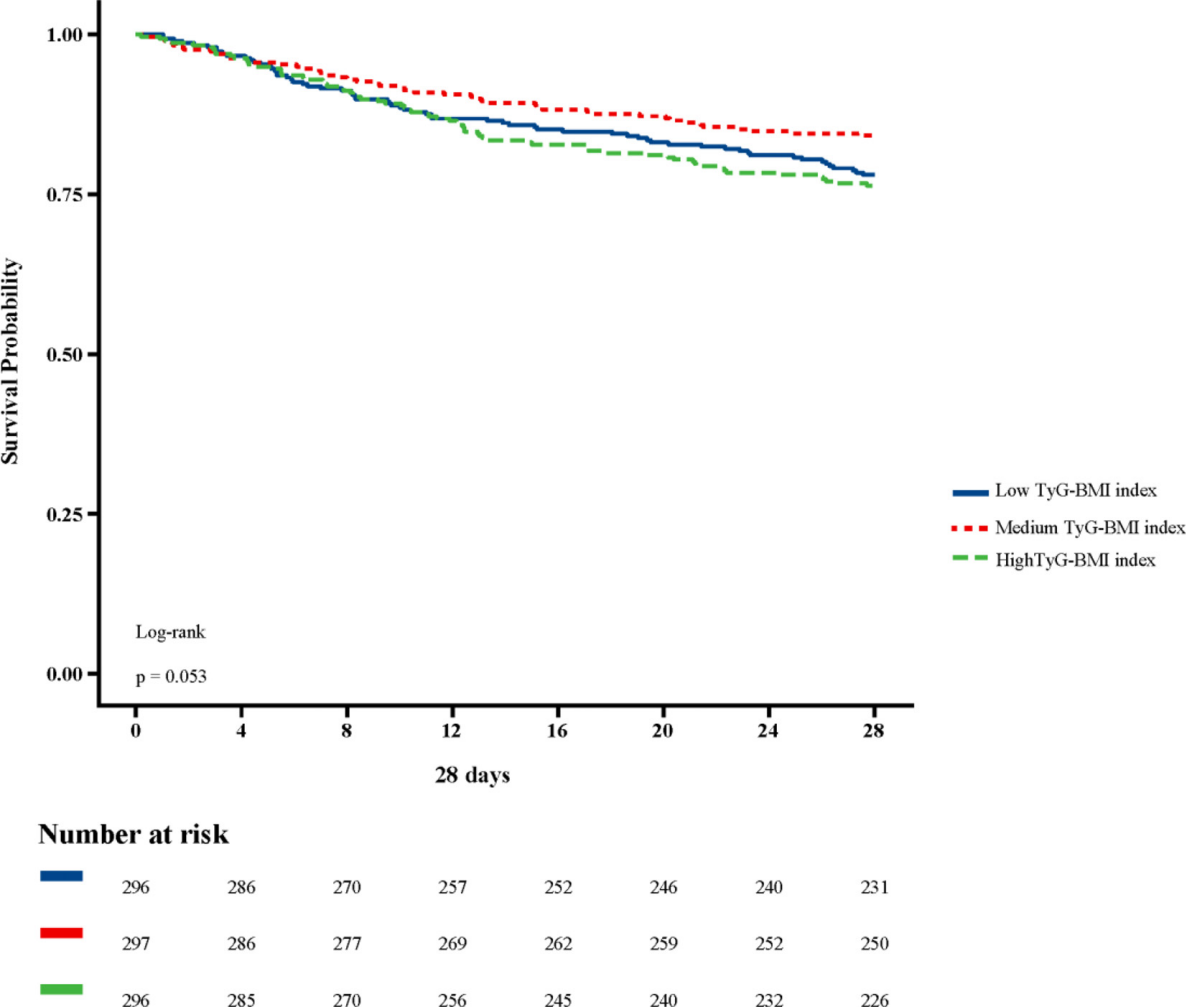
Kaplan−Meier survival curves for 28 days mortality in patients with different TyG-BMI index.

**Figure 4:**
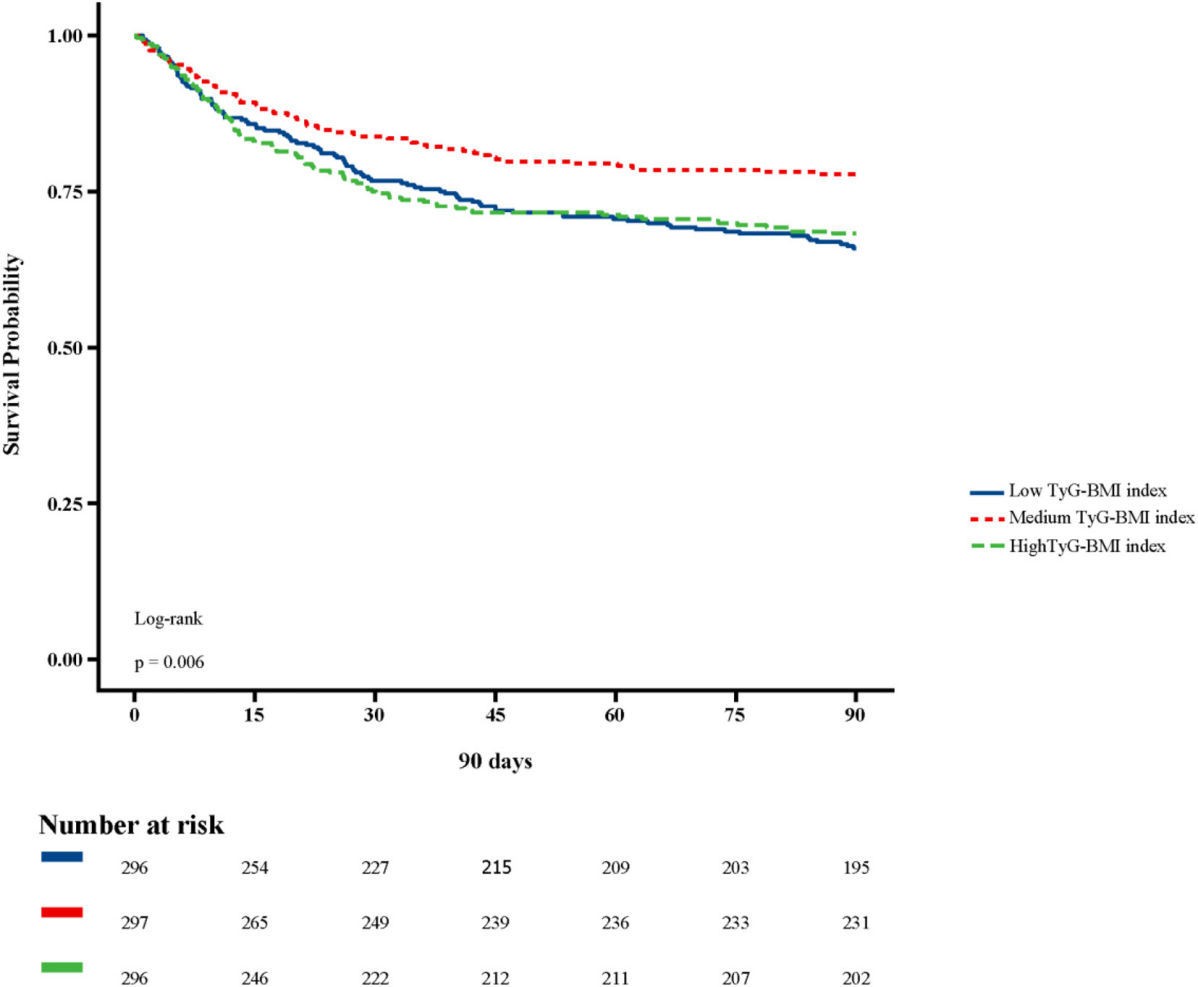
Kaplan−Meier survival curves for 90 days mortality in patients with different TyG-BMI index.

**Figure 5:**
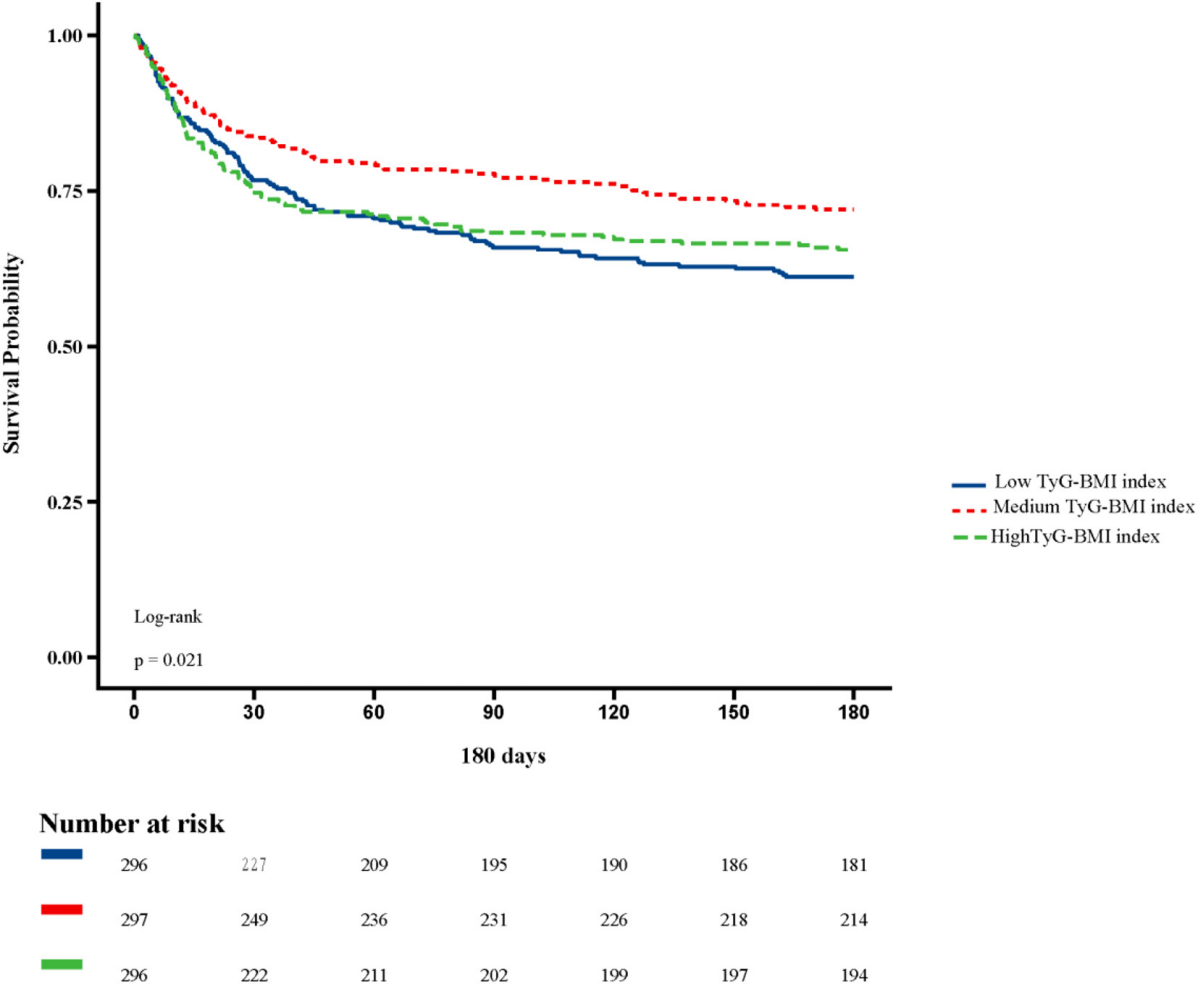
Kaplan−Meier survival curves for 180 days mortality in patients with different TyG-BMI index.

**Figure 6:**
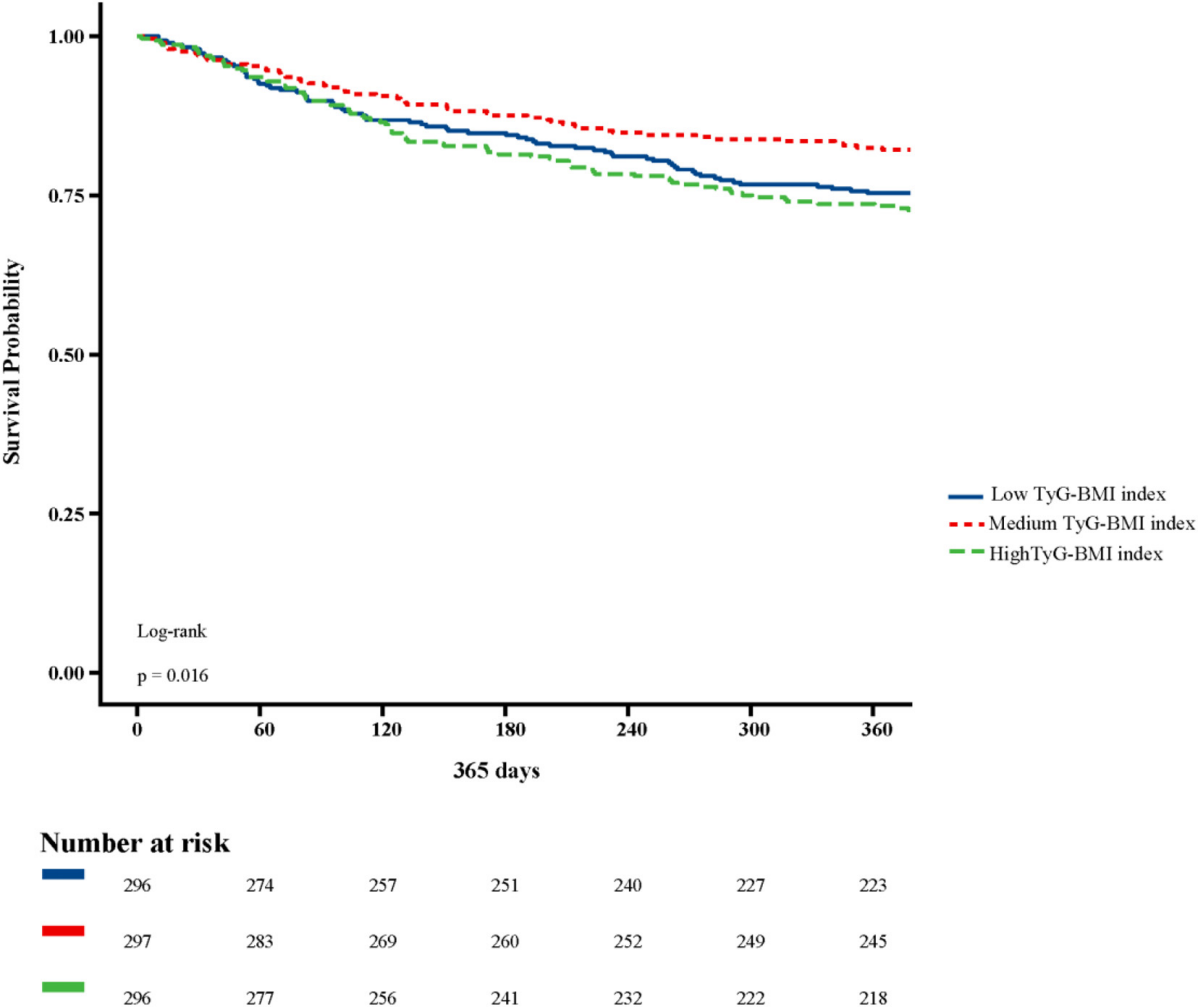
Kaplan−Meier survival curves for 365 days mortality in patients with different TyG-BMI index.

**Figure 2** shows in-hospital and ICU survival rates, with patients in the Medium TyG-BMI index group having the highest in-hospital [245 (82.49%) vs. 232 (78.38%), 223 (75.34%); P=0.102] and ICU survival rates [261 (87.88%) vs. 254 (85.81%), 237 (80.07%); P=0.024], compared to patients in the Low TyG-BMI index and High TyG-BMI index groups. **Figures 3**–**5** demonstrate the prognosis of patients at 28, 90, and 180 days, suggesting that patients in the Medium TyG-BMI index group had the best prognosis. Regarding the primary outcome, which is the 365-day prognosis demonstrated in **Figure 6**, patients in the Medium TyG-BMI index group had the highest 365-day survival rate [201 (67.68%) vs. 166 (56.08%), 188 (63.51%); P=0.013].

### Correlation analysis of 365-day prognosis

To investigate the independent effect of TyG-BMI index on 365-day mortality (**Table 3**), four Cox regression models were applied (low TyG-BMI index was the reference). Model 1, which was not adjusted for factors, had a hazard ratio (HR) of 0.70 (95% CI 0.54- 0.91, P=0.007) for the occurrence of 365-day mortality in the Medium TyG-BMI index group compared to the TyG-BMI index and the High TyG-BMI index groups. Model 2 (adjusted for age, sex) and model 3 (adjusted for Age, Sex, Hypertension, T2DM, T1DM, CKD, AMI, Sroke, WBC, RBC, TC, HDL-C, LDL-C, Bilirubin, Creatinine, TnT, HR, SBP and DBP), found that the HR of 365-day death occurring in the Medium TyG-BMI index group was 0.79 (95%CI 0.60- 1.03, P=0.077) and 0.74 (95%CI 0.57-0.97, P=0.030). Model 4 adjusted for all factors (Age, Sex, Hypertension, T2DM, T1DM, CKD, AMI, Sroke, WBC, RBC, TC, HDL-C, LDL-C, Bilirubin, Creatinine, TnT, HR, SBP, DBP, Sofa, Apsiii, Sirs, Sapsii and Oasis), and found that the HR of 365-day mortality occurring in the Medium TyG-BMI index group was 0.71 (95% CI 0.54-0.93, P=0.012).

**Table 3:** Cox proportional hazard models for 365-day mortality.

	Model 1		Model 2		Model 3		Model 4	
	HR (95% CI)	p-value	HR (95% CI)	p-value	HR (95% CI)	p-value	HR (95% CI)	p-value
Low TyG-BMI index	1.00 (Reference)	–	1.00 (Reference)	–	1.00 (Reference)	–	1.00 (Reference)	–
Medium TyG-BMI index	0.70 (0.54- 0.91)	0.007	0.79 (0.60- 1.03)	0.077	0.74 (0.57-0.97)	0.030	0.71 (0.54-0.93)	0.012
High TyG-BMI index	0.84 (0.65-1.09)	0.188	0.99 (0.75-1.30)	0.919	0.83 (0.63-1.11)	0.219	0.74 (0.56-0.99)	0.049

Model 1: Crude.

Model 2: Adjust: Age, Sex.

Model 3: Adjust: Age, Sex, Hypertension, T2DM, T1DM, CKD, AMI, Sroke, WBC, RBC, TC, HDL-C, LDL-C, Bilirubin, Creatinine, TnT, HR, SBP, DBP.

Model 4: Adjust: Age, Sex, Hypertension, T2DM, T1DM, CKD, AMI, Sroke, WBC, RBC, TC, HDL-C, LDL-C, Bilirubin, Creatinine, TnT, HR, SBP, DBP, Sofa, Apsiii, Sirs, Sapsii, Oasis.

**Figure 7:**
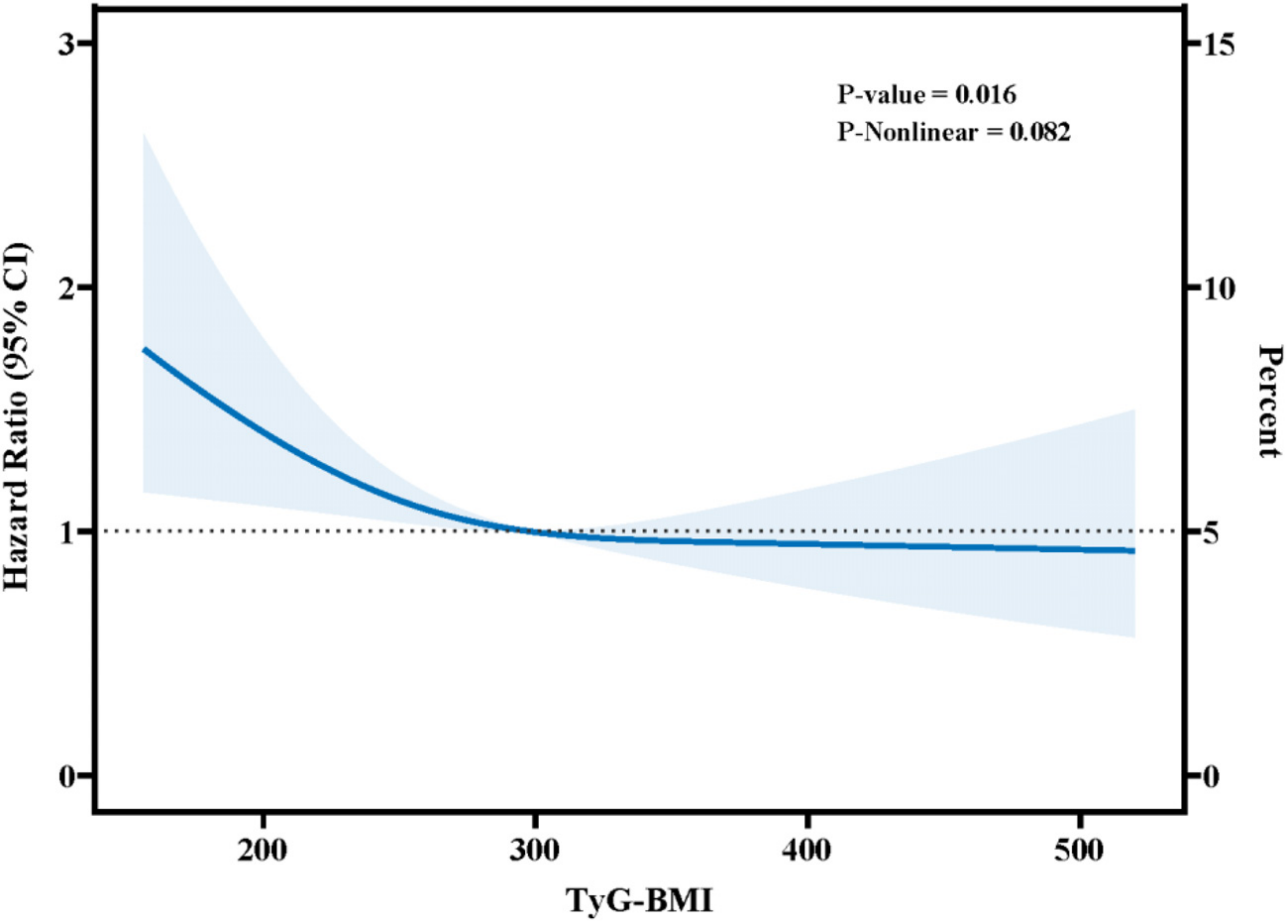
Restricted cubic spline curve for the TyG-BMI index hazard ratio.

RCS curves were performed to explore the nonlinear relationship between TyG-BMI index and 365-day mortality. The RCS curves in the fully adjusted model are illustrated in **Figure 7**, where there is an L-shaped relationship between the TyG-BMI index and the 365-day mortality rate, with a linear decrease in patient mortality as the TyG-BMI index increases (P-Nonlinear=0.082, P=0.016).

### Subgroup analysis

A subgroup analysis assessed the association of TyG-BMI with 365-day mortality outcomes in critically ill patients with CHD (**Table 4**). This study examined the potential impact of the interaction of age, sex, and comorbidities (hypertension, T2DM, T1DM, CKD, AMI, and stroke) on mortality outcomes. However, our analyses did not find any statistically significant association of age, sex and comorbidities with this association. This suggests that the above factors did not have a significant effect on the observed association (p for interaction > 0.05). Our findings suggest that the TyG-BMI index is consistently positively associated with the 365-day risk of death in critically ill patients with CHD in many groups, irrespective of age, sex, and comorbidities.

**Table 4:** Subgroup analysis of TyG-BMI index and 365-day mortality in critically ill patients with CHD.

subgroups	Low TyG-BMI index	Medium TyG-BMI index		High TyG-BMI index		p for interaction
		HR (95%CI)	p-value	HR (95%CI)	p-value	
Sex						0.299
Female	Reference	0.78 (1.10- 1.48)	0.104	1.44 (1.50- 2.48)	0.238	
Male	Reference	1.13 (0.94- 2.04)	0.080	1.81 (1.32- 3.22)	0.065	
Age, years						0.521
Age < 65	Reference	1.47 (1.23-1.48)	0.476	1.17 (1.02-1.38)	0.652	
Age ≥ 65	Reference	1.65 (1.34-1.87)	0.075	1.25 (1.24-2.42)	0.450	
Hypertension						0.920
0	Reference	1.15 (1.94-2.17)	0.098	1.05 (0.96-1.37)	0.725	
1	Reference	1.72 (0.92-1.27)	0.441	1.25 (0.97-1.52)	0.422	
T2DM						0.739
0	Reference	1.32 (1.09-1.42)	0.092	1.22 (1.79-2.02)	0.409	
1	Reference	1.38 (1.12-3.97)	0.206	1.53 (1.22-2.44)	0.087	
T1DM						0.566
0	Reference	1.28 (1.22-1.97)	0.120	1.13 (0.82-1.07)	0.172	
1	Reference	1.19 (0.91-1.64)	0.691	1.21 (1.01-1.14)	0.542	
CKD						0.183
0	Reference	0.92 (1.44-2.87)	0.552	0.71 (1.24-3.81)	0.086	
1	Reference	1.23 (0.96-1.25)	0.053	1.22 (1.06-1.55)	0.062	
AMI						0.056
0	Reference	1.45(1.62-1.80)	0.551	1.01(1.55-2.33)	0.326	
1	Reference	1.66(1.32-2.43)	0.021	1.21(1.22-1.73)	0.540	
Sroke						0.371
0	Reference	1.15(1.02-1.53)	0.290	1.40(1.00-1.13)	0.524	
1	Reference	1.65(0.92-1.27)	0.202	1.53(0.99-1.87)	0.451	

T2DM, Diabetes mellitus type 2; T1DM, Diabetes mellitus type 1; CKD, Chronic kidney disease; AMI, Acute myocardial infarction.

## Discussion

The present study is the first retrospective investigation to explore the association between TyG-BMI index and 365-day mortality in critically ill patients with CHD using the MIMIC IV database.

In our study, we found that medium TyG-BMI index favored the prognosis of patients and suggested an L-shaped relationship between TyG-BMI and 365-day mortality based on the RCS curve. In addition, Cox regression modeling (fully adjusted model) suggested that medium TyG-BMI index was significantly associated with 365-day mortality (HR 0.71, 95%CI 0.54-0.93, P= 0.012). These results may contribute to the development of clinical guidelines to reduce mortality in these patients.

In today’s society, the prevalence of CVD has reached epidemic levels, not only among the general population but also among diagnosed patients with coronary heart disease, where the prevalence has reached astonishing levels (17–19). The relationship between obesity and CVD has been the focus of many epidemiological studies. Commonly, numerous studies have tried to determine the relationship between obesity and the risk of CHD (9). Currently, increasing evidence suggests a significant association between obesity and CVD, especially in the occurrence of coronary heart disease. Zhou (20) et al. proposed that BMI is significantly related to the relative risk (RR) of coronary heart disease, indicating that for every 2-unit increase in BMI, the risk of coronary heart disease may increase by 23% (95% CI: 1.08-1.41). However, some studies have reported that the BMI-mortality curve generally exhibits a U-shape, where mortality from CVD only increases in extreme cases of obesity, highlighting the phenomenon of the obesity paradox (21). This may be due to higher BMI providing better protective effects and sufficient physiological reserves to resist related inflammatory responses in the body. Additionally, there is an incr (22)eased mobilization of endothelial progenitor cells in the obese population, which can promote the regeneration of damaged myocardium and the development of new blood vessels, thus protecting the cardiovascular system from atherosclerosis (23). Whereas IR is strongly associated with obesity (24, 25), with more than 70% of the obese population suffering from IR (26), overweight or obese individuals may be more susceptible to the effects of IR than low or normal weight individuals (27). IR is also a risk factor for the development of CHD, which can lead to increased platelet activity and elevated adhesion-induced expression of thromboxane A2-dependent tissue factor in platelets, leading to thrombosis and inflammation, which in turn may lead to myocardial ischemic events (28). Not only that, IR also causes sympathetic excitation and sodium retention, which aggravate the cardiac load (29). In addition, chronic hyperglycemia and dyslipidemia induced by IR can induce oxidative stress, aggravate inflammatory response, impair endothelial function, and promote the proliferation of smooth muscle cells and collagen deposition, and under the effect of these factors, the patient’s myocardium will be fibrotic, which will ultimately lead to heart failure (30, 31). Undoubtedly, IR poses a risk for the emergence and advancement of CHD. Given the link between IR and obesity, along with its intricate pathophysiology and possible origins, investigating the TyG-BMI index’s impact on CHD patients becomes essential.

IR is not only a core component of metabolic disorders, but also closely associated with dyslipidemia, metabolic syndrome, and the development of atherosclerosis, which together form an important biological basis for the development, progression, and prognosis of CHD. IR is associated with dyslipidemia in the form of hypertriglyceridemia, low high-density lipoprotein cholesterol (HDL-C) levels, and increased numbers of small, dense low-density lipoprotein (LDL) particles, which significantly contribute to the formation of atherosclerosis (32). In addition, IR is strongly associated with multiple risk factors for the metabolic syndrome (e.g., abdominal obesity, atherogenic dyslipidemia, elevated blood pressure, glucose intolerance, and pro-inflammatory and pro-thrombotic states), further exacerbating the risk of cardiovascular disease. IR leads to endothelial dysfunction and cellular damage by exacerbating oxidative stress and inflammatory responses (33, 34). In obese patients, endothelial dysfunction is predominantly characterized by reduced nitric oxide (NO) bioavailability in response to inflammation and oxidative stress, which is a key component in the progression of atherosclerosis (35). The development of atherosclerosis and CHD involves two core processes, inflammation and oxidative stress (36), and IR and obesity are important drivers of these processes. Therefore, IR is inextricably linked to the onset, progression and prognosis of CHD (35, 37). In patients with established ischemic cardiomyopathy, reduced insulin activity limits glucose utilization by cardiomyocytes, forcing a shift to fatty acid metabolism (38). This metabolic shift not only increases myocardial oxygen consumption, but also reduces the compensatory capacity of the non-infarcted myocardium, thus further exacerbating the disease severity of coronary artery disease. The TyG-BMI index, as a comprehensive index combining the TyG index and the BMI, is able to reflect IR and obesity at the same time, and is superior to the single TyG index, the BMI, the conventional lipid levels or other metabolic indicators (14). Therefore, the TyG-BMI index may provide new ideas for risk stratification and individualized treatment of coronary heart disease.

The metabolic abnormalities most closely associated with IR are hyperinsulinemia, some degree of glucose intolerance, hypertriglyceridemia, and low HDL cholesterol concentrations (39). Numerous studies have demonstrated the usefulness and validity of the TyG-BMI index for the assessment of IR (40, 41). In recent investigations, the TyG-BMI index has been shown to be superior to the steady-state model IR index (42). It is important to emphasize that TyG-BMI may be useful as a simple and reliable index for assessing the risk of major adverse cardiovascular events. Cheng et al. found that the TyG-BMI index (per 1 SD) was significantly associated with the occurrence of adverse cardiovascular events in a female population undergoing percutaneous coronary intervention (OR=1.33, 95% CI 1.00-1.76, p = 0.047) (43). In a single-center retrospective study by Liu et al. it was mentioned that the risk of adverse cardiovascular events in patients undergoing percutaneous coronary intervention for ST-segment elevation myocardial infarction increased statistically significantly as the TyG-BMI index increased (p for trend = 0.038) (12). Hu et al. examined the association between the TyG-BMI index and the prognosis of 2509 patients with atrial fibrillation and found an L-shaped association between the TyG-BMI index and 365-day all-cause mortality in critically ill patients with atrial fibrillation (44). These findings suggest that the TyG-BMI index has great value in predicting CVD. Similar results were also found in our study. In the fully adjusted model (model 4), it was shown that critically ill patients with CHD who had a medium TyG-BMI index had a significant advantage in terms of 365-day prognosis, and in the Kaplan−Meier survival curves, it was demonstrated that patients with a medium TyG-BMI index had a better short- term and long-term prognosis. These results may be explained by the fact that the obesity status of patients with higher BMI attenuates the deleterious effects of IR, represented by TyG, in patients with CHD. Perhaps the ‘obesity paradox’ or ‘metabolically healthy obesity’ theory also applies to critically ill CHD patients. On the one hand, obese people have increased endothelial progenitor cells, which promote regeneration of damaged myocardium and formation of new blood vessels, thereby protecting the cardiovascular system from atherosclerosis. In a recent systematic review of cohort studies of patients with CHD, lower mortality from cardiovascular events was observed in moderately obese patients (21). Obese patients may have elevated levels of renin-angiotensin compared to patients with normal BMI, which may also improve long-term cardiovascular prognosis (45). On the other hand, some patients are a subgroup of metabolically healthy obese. Metabolically healthy obesity refers to a subgroup of obese individuals with high BMI associated with a healthy metabolic profile characterized by high insulin sensitivity, a favorable lipid profile and low levels of pro-inflammatory cytokines in plasma and adipose tissue (46, 47). In addition, metabolically healthy obese patients had lower amounts of visceral adipose tissue and liver fat than metabolically unhealthy obese patients (48), and they were at lower risk of cardiovascular events and death, with incidence rates similar to those of normal weight patients (49, 50). In previous studies, it has been found that metabolically healthy obese patients show lower values of intima-media thickness, with evidence in favor of a lower tendency towards an atherosclerotic phenotype (51), allowing myocardial function to be preserved independently of BMI or adiposity. This is why in our study we found that patients with medium TyG-BMI index had better outcomes compared to other patients. Considering the interaction of TG, Glucose, and BMI, the predictive value of the TyG-BMI index for the risk of adverse outcomes needs to focus on the combined effect of these three factors. In our study, the TyG-BMI index may prove to better reveal their interactions and synergistic effects, thus more accurately predicting the risk of death in patients with CHD, which proved to have a significant L-shaped relationship with 365-day mortality.

### Limitation

It has to be recognized that our study has some limitations. First, this was a single-center retrospective study based on observational data extracted from the MIMIC-IV database, which made it difficult to establish a definitive causal relationship. Second, we were unable to trace back the missing data from the database, which led us to remove some of the patients with missing data, which resulted in a moderate sample size and required the results of a large sample size cohort study to support our findings. Third, we were unable to compare the TyG-BMI index with other current IR measurement techniques. Fourth, extracting blood glucose and lipid data as the first measurements of patients admitted to the ICU did not fully determine whether these measurements were derived from fasting patients. In addition, we were unable to elucidate the time of CHD onset and the primary cause of death, which would have reduced the clinical relevance of the current analysis.

## Conclusion

We found an L-shaped association between the TyG-BMI index and 365-day all-cause mortality in critically ill patients with CHD. As the TyG-BMI index increased, the mortality rate of patients tended to decrease linearly. The TyG-BMI index may serve as an effective tool for classifying and preventing the risk of developing CHD. In addition, given the obesity paradox, BMI should be of equal concern in patients with a definite diagnosis of CHD.

## Data Availability

The raw data supporting the conclusions of this article will be made available by the authors, without undue reservation.

## References

[1] Falk E. Pathogenesis of atherosclerosis. J Am Coll Cardiol. (2006) 47:C7–C12. doi: 10.1016/j.jacc.2005.09.068 16631513

[2] Poznyak AV Bharadwaj D Prasad G Grechko AV Sazonova MA Orekhov AN. Renin-angiotensin system in pathogenesis of atherosclerosis and treatment of CVD. Int J Mol Sci. (2021) 22:6702. doi: 10.3390/ijms22136702 34206708 PMC8269397

[3] Cui H Liu Q Wu Y Cao L. Cumulative triglyceride-glucose index is a risk for CVD: a prospective cohort study. Cardiovasc Diabetol. (2022) 21:22. doi: 10.1186/s12933-022-01456-1 35144621 PMC8830002

[4] Zhao D Liu J Wang M Zhang X Zhou M. Epidemiology of cardiovascular disease in China: current features and implications. Nat Rev Cardiol. (2019) 16:203–12. doi: 10.1038/s41569-018-0119-4 30467329

[5] Vaduganathan M Mensah GA Turco JV Fuster V Roth GA. Global burden of cardiovascular diseases and risk factors, 1990–2019. J Am Coll Cardiol. (2020) 76:2982–3021. doi: 10.1016/j.jacc.2022.11.005 33309175 PMC7755038

[6] Luo E Wang D Yan G Qiao Y Liu B Hou J. High triglyceride–glucose index is associated with poor prognosis in patients with acute ST-elevation myocardial infarction after percutaneous coronary intervention. Cardiovasc Diabetol. (2019) 18:150. doi: 10.1186/s12933-019-0957-3 31722708 PMC6852896

[7] Lebovitz HE. Insulin resistance: definition and consequences. Exp Clin Endocrinol Diabetes. (2001) 109 Suppl 2:S135–48. doi: 10.1055/s-2001-18576 11460565

[8] Huang Y Cai X Mai W Li M Hu Y. Association between prediabetes and risk of cardiovascular disease and all cause mortality: systematic review and meta-analysis. BMJ. (2016) 355:i5953. doi: 10.1136/bmj.i5953 27881363 PMC5121106

[9] Katta N Loethen T Lavie CJ Alpert MA. Obesity and coronary heart disease: epidemiology, pathology, and coronary artery imaging. Curr Problems Cardiol. (2021) 46:100655. doi: 10.1089/met.2008.0034 32843206

[10] Simental-Mendía LE Rodríguez-Morán M Guerrero-Romero F. The product of fasting glucose and triglycerides as surrogate for identifying insulin resistance in apparently healthy subjects. Metab Syndrome Related Disord. (2008) 6:299–304. doi: 10.1097/MCA.00000000000001242 19067533

[11] Drwiła-Stec D Rostoff P Gajos G. Predictive value of metabolic score for insulin resistance and triglyceride glucose-BMI among patients with acute myocardial infarction in 1-year follow-up. Coronary Artery Dis. (2023) 34:314–9. doi: 10.1097/MCA.00000000000001242 PMC10758348 37222212

[12] Liu M Pan J Meng K Wang Y Sun X Ma L. Triglyceride-glucose body mass index predicts prognosis in patients with ST-elevation myocardial infarction. Sci Rep. (2024) 14(1):976. doi: 10.1038/s41598-023-51136-7 38200157 PMC10782013

[13] Alizargar J Bai C Hsieh N Wu SV. Use of the triglyceride-glucose index (TyG) in cardiovascular disease patients. Cardiovasc Diabetol. (2020) 19:8. doi: 10.1186/s12933-019-0982-2 31941513 PMC6963998

[14] Er L Wu S Chou H Hsu LA Teng MS Sun YC. Triglyceride glucose-body mass index is a simple and clinically useful surrogate marker for insulin resistance in nondiabetic individuals. PloS One. (2016) 11:e0149731. doi: 10.1371/journal.pone.0149731 26930652 PMC4773118

[15] Brody GH Yu T Chen E Ehrlich KB Miller GE. Racial discrimination, body mass index, and insulin resistance: A longitudinal analysis. Health Psychol. (2018) 37:1107–14. doi: 10.1037/hea0000674 PMC6277234 30307274

[16] Khan SH Sobia F Niazi NK Manzoor SM Fazal N Ahmad F. Metabolic clustering of risk factors: evaluation of Triglyceride-glucose index (TyG index) for evaluation of insulin resistance. Diabetol Metab Syndrome. (2018) 10:74. doi: 10.1186/s13098-018-0376-8 PMC6173832 30323862

[17] Mclellan F. Obesity rising to alarming levels around the world. Lancet (British edition). (2002) 359:1412. doi: 10.1016/S0140-6736(02):08397-6 11978348

[18] Kenchaiah S Evans JC Levy D Wilson PW Benjamin EJ Larson MG. Obesity and the risk of heart failure. New Engl. (2002) 347:305–13. doi: 10.1056/NEJMoa020245 12151467

[19] Lakka HM Lakka TA Tuomilehto J Salonen JT. Abdominal obesity is associated with increased risk of acute coronary events in men. Eur Heart J. (2002) 23:706–13. doi: 10.1053/euhj.2001.2889 11977996

[20] Zhou B Wu Y Yang J Li Y Zhang H Zhao L. Overweight is an independent risk factor for cardiovascular disease in Chinese populations. Obes Rev. (2002) 3:147–56. doi: 10.1046/j.1467-789X.2002.00068.x 12164466

[21] Romero-Corral A Montori VM Somers VK Korinek J Thomas RJ Allison TG. Association of bodyweight with total mortality and with cardiovascular events in coronary artery disease: a systematic review of cohort studies. Lancet. (2006) 368:666–78. doi: 10.1016/S0140-6736(06)69251-9 16920472

[22] Tb H Gc F Al C. Obesity and the obesity Paradox in Heart failure. Prog Cardiovasc Dis. (2018) 61:151–6. doi: 10.1016/j.pcad.2018.05.005 29852198

[23] Biasucci LM Graziani F Rizzello V Liuzzo G Guidone C De Caterina AR. Paradoxical preservation of vascular function in severe obesity. Am J Med. (2010) 123:727–34. doi: 10.1016/j.amjmed.2010.02.016 20670727

[24] Lteif AA Han K Mather KJ. Obesity, insulin resistance, and the metabolic syndrome. Circulation. (2005) 112:32–8. doi: 10.1161/CIRCULATIONAHA.104.520130 15983246

[25] Abbasi F Brown BW Lamendola C McLaughlin T Reaven GM. Relationship between obesity, insulin resistance, and coronary heart disease risk. J Am Coll Cardiol. (2002) 40:937–43. doi: 10.1016/S0735-1097(02)02051-X 12225719

[26] Calori G Lattuada G Piemonti L Garancini MP Ragogna F Villa M. Prevalence, metabolic features, and prognosis of metabolically healthy obese italian individuals. Diabetes Care. (2011) 34:210–5. doi: 10.2337/dc10-0665 PMC3005463 20937689

[27] Xu J Wang A Meng X Jing J Wang Y Wang Y. Obesity-stroke paradox exists in insulin-resistant patients but not insulin sensitive patients. Stroke. (2019) 50:1423–9. doi: 10.1161/STROKEAHA.118.023817 31043152

[28] Gerrits AJ Koekman CA Van Haeften TW Akkerman JW. Platelet tissue factor synthesis in type 2 diabetic patients is resistant to inhibition by insulin. Diabetes. (2010) 59:1487–95. doi: 10.2337/db09-1008 PMC2874710 20200314

[29] Da Silva AA Do Carmo JM Li X Wang Z Mouton AJ Hall JE. Role of hyperinsulinemia and insulin resistance in hypertension: metabolic syndrome revisited. Can J Cardiol. (2020) 36:671–82. doi: 10.1016/j.cjca.2020.02.066 PMC7219403 32389340

[30] Zhou MS Schulman IH Zeng Q. Link between the renin-angiotensin system and insulin resistance: implications for cardiovascular disease. . Vasc Med. (2012) 17:330–41. doi: 10.1177/1358863X12450094 22814999

[31] Hill MA Yang Y Zhang L Sun Z Jia G Parrish AR. Insulin resistance, cardiovascular stiffening and cardiovascular disease. Metabolism: Clin Exp. (2021) 119:154766. doi: 10.1016/j.metabol.2021.154766 33766485

[32] Powell-Wiley TM Poirier P Burke LE Després JP Gordon-Larsen P Lavie CJ. Obesity and cardiovascular disease: A scientific statement from the american heart association. Circulation. (2021) 143(21):e984–e1010. doi: 10.1161/CIR.0000000000000973 PMC8493650 33882682

[33] Achari A Jain S. Adiponectin, a therapeutic target for obesity, diabetes, and endothelial dysfunction. Int J Mol Sci. (2017) 18:1321. doi: 10.3390/ijms18061321 28635626 PMC5486142

[34] Engin A. Endothelial dysfunction in obesity. Adv Exp Med Biol. (2017) 960:345. doi: 10.1007/978-3-319-48382-5 28585207

[35] Ormazabal V Nair S Elfeky O Aguayo C Salomon C Zuñiga FA. Association between insulin resistance and the development of cardiovascular disease. Cardiovasc Diabetol. (2018) 17(1):122. doi: 10.1186/s12933-018-0762-4 30170598 PMC6119242

[36] Arenas De Larriva AP Limia-Pérez L Alcalá-Díaz JF Alonso A López-Miranda J Delgado-Lista J. Ceruloplasmin and coronary heart disease—A systematic review. Nutrients. (2020) 12:3219. doi: 10.3390/nu12103219 33096845 PMC7589051

[37] Othman EM Leyh A Stopper H. Insulin mediated DNA damage in mammalian colon cells and human lymphocytes *in vitro* . Mutat Research/fundamental Mol Mech Mutagenesis. (2013) 745-746:34–9. doi: 10.1016/j.mrfmmm.2013.03.006 23524287

[38] Tao L Xu J Wang T Hua F Li JJ. Triglyceride-glucose index as a marker in cardiovascular diseases: landscape and limitations. Cardiovasc Diabetol. (2022) 21:68. doi: 10.1186/s12933-022-01511-x 35524263 PMC9078015

[39] Gm R. Role of insulin resistance in human disease. Diabetes. (1988) 37:1595–607. doi: 10.2337/diab.37.12.1595 3056758

[40] Jiang C Yang R Kuang M Yu M Zhong M Zou Y. Triglyceride glucose-body mass index in identifying high-risk groups of pre-diabetes. Lipids Health Dis. (2021) 20:161. doi: 10.1186/s12944-021-01594-7 34774061 PMC8590771

[41] Wang M Chang M Shen P Wei W Li H Shen G. Application value of triglyceride-glucose index and triglyceride-glucose body mass index in evaluating the degree of hepatic steatosis in non-alcoholic fatty liver disease. Lipids Health Dis. (2023) 22:186. doi: 10.1186/s12944-023-01954-5 37924128 PMC10623715

[42] Lim J Kim J Koo SH Kwon GC. Comparison of triglyceride glucose index, and related parameters to predict insulin resistance in Korean adults: An analysis of the 2007-2010 Korean National Health and Nutrition Examination Survey. PloS One. (2019) 14:e0212963. doi: 10.1371/journal.pone.0212963 30845237 PMC6405083

[43] Cheng Y Fang Z Zhang X Wen Y Lu J He S. Association between triglyceride glucose-body mass index and cardiovascular outcomes in patients undergoing percutaneous coronary intervention: a retrospective study. Cardiovasc Diabetol. (2023) 22(1):75. doi: 10.1186/s12933-023-01794-8 36997935 PMC10064664

[44] Hu Y Zhao Y Zhang J Li C. The association between triglyceride glucose-body mass index and all-cause mortality in critically ill patients with atrial fibrillation: a retrospective study from MIMIC-IV database. Cardiovasc Diabetol. (2024) 23(1):64. doi: 10.1186/s12933-024-02153-x 38341579 PMC10859027

[45] Novo G Guttilla D Fazio G Cooper D Novo S. The role of the renin–angiotensin system in atrial fibrillation and the therapeutic effects of ACE-Is and ARBS. Br J Clin Pharmacol. (2008) 66:345–51. doi: 10.1111/j.1365-2125.2008.03234.x PMC2526238 18782141

[46] Primeau V Coderre L Karelis AD Brochu M Lavoie ME Messier V. Characterizing the profile of obese patients who are metabolically healthy. Int J Obes (Lond). (2011) 35:971–81. doi: 10.1038/ijo.2010.216 20975726

[47] Naukkarinen J Heinonen S Hakkarainen A Lundbom J Vuolteenaho K Saarinen L. Characterising metabolically healthy obesity in weight-discordant monozygotic twins. Diabetologia. (2014) 57(1):167–76. doi: 10.1007/s00125-013-3066-y 24100782

[48] Stefan N Kantartzis K Machann J Schick F Thamer C Rittig K. Identification and characterization of metabolically benign obesity in humans. Arch Internal Med. (2008) 168:1609–16. doi: 10.1001/archinte.168.15.1609 18695074

[49] Ogorodnikova AD Kim M Mcginn AP Muntner P Khan U Wildman RP. Incident cardiovascular disease events in metabolically benign obese individuals. Obesity. (2012) 20:651–9. doi: 10.1038/oby.2011.243 PMC3494999 21799477

[50] Appleton SL Seaborn CJ Visvanathan R Hill CL Gill TK Taylor AW. Diabetes and cardiovascular disease outcomes in the metabolically healthy obese phenotype. Diabetes Care. (2013) 36:2388–94. doi: 10.2337/dc12-1971 PMC3714523 23491523

[51] Dobson R Burgess MI Sprung Irwin A Hamer M Jones J. Metabolically healthy and unhealthy obesity: differential effects on myocardial function according to metabolic syndrome, rather than obesity. Int J Obes. (2016) 40:153–61. doi: 10.1038/ijo.2015.151 26271188

